# Rapid uptake and slow depuration: Health risks following cyanotoxin accumulation in mussels?^☆^

**DOI:** 10.1016/j.envpol.2020.116400

**Published:** 2021-02-15

**Authors:** Dolores Camacho-Muñoz, Julia Waack, Andrew D. Turner, Adam M. Lewis, Linda A. Lawton, Christine Edwards

**Affiliations:** a School of Pharmacy and Life Sciences, Robert Gordon University, Aberdeen, AB10 7GJ, UK; b Centre for Environment, Fisheries and Aquaculture Science, Barrack Road, The Nothe, Weymouth, Dorset, DT4 8UB, UK

**Keywords:** Hepatotoxins, Brackish water, Bivalves, Human health

## Abstract

Freshwater cyanobacteria produce highly toxic secondary metabolites, which can be transported downstream by rivers and waterways into the sea. Estuarine and coastal aquaculture sites exposed to toxic cyanobacteria raise concerns that shellfish may accumulate and transfer cyanotoxins in the food web. This study aims to describe the competitive pattern of uptake and depuration of a wide range of microcystins (MC-LR, MC-LF, MC-LW, MC-LY, [Asp3]-MC-LR/[Dha7]-MC-LR, MC-HilR) and nodularins (NOD cyclic and linear) within the common blue mussel *Mytilus edulis* exposed to a combined culture of *Microcystis aeruginosa* and *Nodularia spumigena* into the coastal environment.

Different distribution profiles of MCs/NODs in the experimental system were observed. The majority of MCs/NODs were present intracellularly which is representative of healthy cyanobacterial cultures, with MC-LR and NOD the most abundant analogues. Higher removal rate was observed for NOD (≈96%) compared to MCs (≈50%) from the water phase. Accumulation of toxins in *M. edulis* was fast, reaching up to 3.4 μg/g shellfish tissue four days after the end of the 3-days exposure period, with NOD (1.72 μg/g) and MC-LR (0.74 μg/g) as the dominant toxins, followed by MC-LF (0.35 μg/g) and MC-LW (0.31 μg/g). Following the end of the exposure period depuration was incomplete after 27 days (0.49 μg/g of MCs/NODs). MCs/NODs were also present in faecal material and extrapallial fluid after 24 h of exposure with MCs the main contributors to the total cyanotoxin load in faecal material and NOD in the extrapallial fluid. Maximum concentration of MCs/NODs accumulated in a typical portion of mussels (20 mussels, ≈4 g each) was beyond greater the acute, seasonal and lifetime tolerable daily intake. Even after 27 days of depuration, consuming mussels harvested during even short term harmful algae blooms in close proximity to shellfish beds might carry a high health risk, highlighting the need for testing.

## Highlights

• MCs/NODs were present in *M. edulis* tissue, faecal material and extrapallial fluid after 24 h of exposure.
• Depuration of MCs/NODs was incomplete after 27 days.
• MCs/NODs distributed differently in the experimental system.
• Consumption of *M. edulis* harvested after a short term HAB can pose a human and animal health risk.

## Graphical abstract

**Figure:**
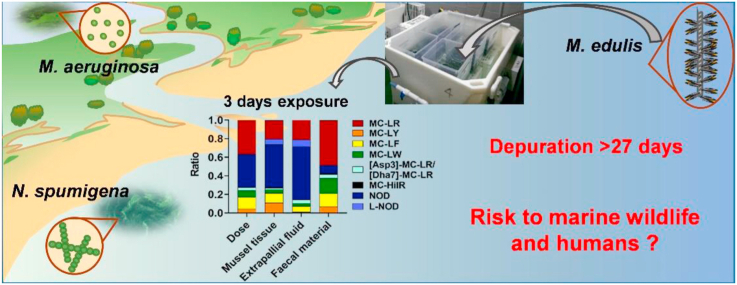

## Teaser

Accumulation of freshwater cyanotoxins in marine mussels is fast but depuration is slow what could result in high health risk for wildlife and humans, highlighting the need for testing.

## 1 Introduction

The combination of anthropogenic activity and climate change have led to a significant increase in the occurrence of freshwater harmful algal blooms (HABs) across the globe, frequently adding additional stress to already compromised water supplies (Carmichael and Boyer, 2016; Visser et al., 2016). Of particular concern is that many of the cyanobacterial species responsible for these blooms produce an array of highly toxic secondary metabolites which can severely impact human and animal health along with ecosystem function (Ferrão-Filho and Kozlowsky-Suzuki, 2011; Svirčev et al., 2017).

Globally the most commonly occurring freshwater cyanotoxins are microcystins (MCs) (Díez-Quijada et al., 2019; Rastogi et al., 2014), a large family (246 reported variants) (Spoof and Catherine, 2017) of cyclic heptapeptides with well documented acute and chronic toxicity (Ferrão-Filho and Kozlowsky-Suzuki, 2011; Svirčev et al., 2017). MCs are produced by several cyanobacteria genera, including the planktonic *Microcystis, Planktothrix, Anabaena* species, and the benthic *Oscillatoria* among others (Rastogi et al., 2014). Nodularin (NOD) is also a cyclic peptide containing five amino acids only produced by the species *Nodularia spumigena* mainly found in brackish water (Lehtimäki et al., 2000). MCs and NOD are potent hepatotoxins, cytotoxins, neurotoxins and endotoxins (Funari and Testai, 2008) and the common side chain Adda is essential for toxicity (Fig. S1) (Akter et al., 2016). The mechanism of action is related to the inhibition of protein serine/threonine phosphatases (PP2A and PP1) resulting in hyperphosphorylation in the cells and inducing morphological changes on the cytoskeleton causing cellular breakdown and death (Funari and Testai, 2008; Kuiper-Goodman et al., 1999). MCs and NOD also induce cellular toxicity in the liver. Acute toxicity lead to gastroenteritis, liver damage, jaundice and neurotoxic effects (WHO, 2017), and even death. At sub-lethal doses they are potent liver tumour promoters and produce oxidative DNA damage and apoptosis (Funari and Testai, 2008; Kuiper-Goodman et al., 1999).

The World Health Organization (WHO) has set a recommended maximum allowable level in drinking water of 1 μg/L for MC-LR (WHO, 2017), the most reported of the MCs, and the Environmental Protection Agency of the USA at 10-day health advisory of 1.6 μg/L for MCs for adults (USEPA, 2015).

Human exposure to these cyanotoxins may occur through ingestion of contaminated drinking water, consumption of contaminated fish and vegetables, or through exposure to water bodies with cyanobacterial blooms during recreational activities (Flores et al., 2018; Lehtimäki et al., 2011; Svirčev et al., 2019).

Knowledge and understanding of hepatotoxins in the marine environment is still limited, with initial reports of MCs in mussels and implication as the cause of a severe liver disease in Atlantic salmon in aquaculture sites of the coast of Canada in the early 90s (Andersen et al., 1993). However, the source of the MCs was not identified and assumed to be of bacterial origin. Only in the last 20 years there has been an increase in reports of MCs and NOD in the marine environment (Carlsson and Rita, 2019; Ferrão-Filho and Kozlowsky-Suzuki, 2011; Mazur-Marzec et al., 2007; Miller et al., 2010; Peacock et al., 2018; Vareli et al., 2012; Zhang et al., 2009). It has been suggested that some marine species are capable of MC production as evidenced in a Greek study where MC-LR and MC-YR were detected in *M. galloprovincialis* where the cyanobacterial population was dominated by *Synechococcus* and *Synechocystsis* (Vareli et al., 2012). Even though levels in water (0.003–19.8 ng/L) were below the WHO limit of 1 μg/L, field samples of *M. galloprovincialis* contained MCs at concentrations up to 142 μg/kg which exceeded the upper limit of the tolerable daily intake (TDI) determined by WHO (0.04 μg/kg body weight, or 3 μg for an adult weighting 75 kg) (WHO, 2017).

Currently a significant threat to estuaries and coastal environments is from transportation of freshwater toxic cyanobacterial blooms (Peacock et al., 2018; Preece et al., 2017; Tatters et al., 2017). These situations are frequent in certain regions e.g. San Francisco Bay, USA (Lehman et al., 2005, 2020; Peacock et al., 2018); Chesapeake Bay, USA (Tango and Butler, 2008); James River Estuary, USA (Bukaveckas et al., 2018) and Isahaya and Ariake Bay, Japan (Umehara et al., 2017). Tango and Butler (2008) reported maximum MCs levels of 658 μg/L in water samples from Chesapeake Bay which most likely originated from a *Microcystis* bloom in the Sassafras River. Exposure to toxic cyanobacteria can negatively impact high trophic levels (Brown et al., 2018; Miller et al., 2010). One of the most serious examples involved the death of 21 sea otters following ingestion of MCs contaminated seafood for which the likely MCs source was a *Microcystis* bloom, present in Lake Pinto that had been transported into the coastal area via the Pajaro River (Miller et al., 2010). Further evidence for this transfer was found in blue crabs (up to 105 μg/kg MC-LR in muscle tissue) (Garcia et al., 2010), dolphins (1.3–19.9 μg/kg dw total MCs/NODs in liver) (Brown et al., 2018), and fish for human consumption (0.5–1917 μg/kg in muscle tissue and 4.5–215.2 μg/kg whole fish) (Poste et al., 2011).

A recent publication by the European Food Standard Authority has pointed out that the potential of estuarine and coastal aquaculture facilities being exposed to cyanotoxins remains a global concern and represent a rising hazard for the general public (Testai et al., 2016). Shellfish are main vectors of these toxins in the marine trophic chain. Whilst it is well known that MCs/NOD accumulate rapidly in mussels, competitive exposure of mussels with freshwater cyanobacteria, *Microcystis* and brackish water *Nodularia* was evaluated to mimic freshwater contamination of important coastal regions for aquaculture. This study aims to describe the pattern of uptake and depuration of a wide range of MCs/NODs within the mussels (*Mytilus edulis)* during a 3-day exposure period, to resemble a natural and brackish HAB inputting into the coastal environment, to a combined culture of *Microcystis aeruginosa* and *Nodularia spumigena* culture followed by 27-day depuration period and to assess the MCs/NODs mass balance under controlled laboratory conditions. The data collected in this study would further help to build a platform of knowledge for risk assessments.

## 2 Materials and methods

### 2.1 Chemicals and reagents

LC-MS grade acetonitrile, water and formic acid and HPLC-grade methanol and water were purchased from Fisher (ThermoFisher, UK). Reference toxin standards of microcystins (MC-LR, MC-RR, MC-YR, MC-WR, MC-LW, MC-LA, MC-LY, MC-LF, MC-HtyR, MC-HiLR, [Asp3]-MC-LR/[Dha7]-MC-LR) and NOD ≥95% were as per Enzo Life Sciences (Exeter, UK) (Fig. S1). A certified standard of [Dha7]-MC-LR and a pre-certified freeze-dried matrix reference material of blue-green algae (RM-BGA, Lot 201301) containing a range of MCs were purchased from the Institute of Biotoxin Metrology, National Research Council Canada (Ontario, Canada).

A mixed stock solution was prepared by combining aliquots of each toxin to give a final concentration of 327 μg/L. For external calibration, a seven point calibration curve was prepared by serial dilution with methanol/water (1:1, v/v) in the range of 0.33–327 μg/L for each toxin and stored at – 18 °C. A quality control reference material (RM-BGA, National Research Council, Halifax, Canada) was prepared, with toxins extracted in the supernatant after 28 mL of methanol/water (1:1, v/v) + 0.1% acetic acid were added to RM-BGA (280 mg) and subsequent centrifugation (4500 ×*g*; 10 min).

Shellfish diet 1800 (approximately 7.4 × 10^11^ cells/mL) was purchased from ReedMariculture Inc., (US) and dilutions were made in water/seawater (10:0.86, v/v).

### 2.2 Culturing of *N. spumigena* and *M. aeruginosa*

*N. spumigena* KAC 66 (Kalmar Algae Collection, Kalmar, Sweden) is a filamentous nitrogen fixing cyanobacteria and *M. aeruginosa* PCC 7813 (Pasteur Culture Collection of Cyanobacteria, Paris, France) is unicellular in laboratory cultures. Both cultures were grown in modified BG-11 medium with 75 g/L sodium nitrate (Stanier et al., 1971). In addition, media for *N. spumigena* KAC 66 was supplemented with 20% (w/v) instant ocean salt (Aquarium Systems Inc., Sarrebourg, France). Cultures were maintained at 20–23 °C, under continuous illumination (10–15 μmol/m^2^/s^1^) and sparged with sterile air at 2.3 L/min. To obtain homogeneous cells, four days before harvesting sparging was stopped. *N. spumigena* cells concentrated at the surface due to the presence of intracellular gas vesicles whereas *M. aeruginosa* cells settled at the bottom. Cells were collected and transferred into 10 L carboys for transportation to the test site where they were maintained in 25 L carboys at 17 ± 1 °C with mild aeration and a light cycle of 17 h illumination (24 μmol/m^2^/s^1^) and 7 h darkness. Cultures were 4 weeks old at the beginning of the experiment. Cell densities were monitored by optical density at 730 nm.

### 2.3 Accumulation and depuration of cyanotoxins in *Mytilus edulis*

Live mussels (shell length 50–65 mm) sourced from the Shetland Islands (UK) were acclimatised to laboratory tank conditions for a week and cleaned of barnacles and other debris prior to the experiment (Waack, 2017).

Two tanks (300 L) were filled with ≈150 L of seawater, maintained at 16 ± 1 °C and equipped with ultraviolet sterilisers (class 1 IP64, twin UV 24 W, 240 V, 50 Hz, Tropical Marine Centre, UK). One tank was used for the exposure of *M. edulis* (n = 420) to *M. aeruginosa* (3.9 × 10^6^ cells/L final concentration) and *N. spumigena* (3.1 × 10^6^ cells/L final concentration) and the second tank (n = 420 mussels) was used for negative control. Six containers (12 L; sub-container A (1–2), B (1–2) and C (1–2) filled with 10 L of 10.13039/100008457UV sterilised, filtered seawater (≈35 psu), housing 70 mussels supported by mesh baskets were placed in each container (Fig. S2 and S3). Gentle aeration was provided via PVC tubing (4.00 mm bore size, 1.00 mm wall thickness).

The exposure period lasted 3 days (Fig. S2 and S3). Mussels were exposed daily to ≈47 μg/L of NOD and ≈85 μg/L of MCs congeners (≈48 μg/L of MC-LR), from a combined culture of *N. spumigena* (300 mL) and *M. aeruginosa* (400 mL), added to the containers. The concentrations selected of MCs and NOD represent realistic values in line with reports of cyanotoxins occurring in natural HABs and other studies on accumulation of cyanotoxins in the food web (Buratti et al., 2017; Peacock et al., 2018; Preece et al., 2017; Umehara et al., 2017). The mussels were fed daily (0.14 mL of shellfish diet diluted into 500 mL of water/seawater mixture (10:0.86, v/v). The water was renewed daily (after 21 h post-feeding). At the end of the exposure period a depuration period of 27 days started. During the depuration period mussels were fed daily (0.33 mL of shellfish diet diluted into 1.2 L of water/seawater mixture (10:0.86, v/v) and the water was renewed daily (after 21 h, post-feeding). The negative control tank ran under the depuration period conditions for 30 days.

The decrease in toxin concentration in the water tank post feeding was monitored every 21 h during the exposure period by withdrawal of water samples (100 mL) from each container.

#### 2.3.1 Sampling

Cyanobacteria feed stock cultures were sampled (1 mL) prior to feeding (day 1–3) to assess intracellular and extracellular content of cyanotoxins (Fig. S3) (Waack, 2017).

To accommodate the lengthy depuration period (27 days), two experimental containers (sub-containers A1 and A2, B1 and B2 and C1 and C2; n = 70 mussels each sub-container; Fig. S3) were treated as one overall sample of 140 mussels from which 10 mussels were sampled from container A, B and C (e.g. 5 mussels from A1 and 5 mussels from A2; Fig. S3). Mussels (n = 10) were randomly collected and sacrificed after 1, 2 and 3 days of exposure and after 2, 4, 6, 9, 12, 15, 18, 21, 24 and 27 days of depuration. For each mussel sampled a naïve “stunt double” mussel, was placed into a separate mesh basket to maintain a constant ratio of mussels throughout the experiment (Amorim and Vasconcelos, 1999).

Water samples (200 mL; combined sample from sub-container 1 (100 mL) and sub-container 2 (100 mL)) were taken twice daily throughout the experiment: post feeding (t_0h_) and prior to the daily water exchange (t_21h_) (Fig. S3). Mussels were taken out of the containers prior sampling and then they were placed back in to ensure identical t_0h_.

Faecal material was collected daily throughout the experiment before the daily water renewal. Remaining water from sub-containers 1 and 2 were combined (approx. 22 L), mixed and filtered (80 μm steel mesh) (Fig. S3). Collected faecal material was washed out from the filter and its weight recorded.

### 2.4 Extraction of toxins

#### 2.4.1 Extraction of toxins from feed stock cultures

Optimization of solvent composition for toxin extractions were described previously (Turner et al., 2018). Feed stock culture samples (1 mL) were centrifuged (12,470 × *g*, 10 min; Sigma 1–14 K, Osterode, Germany) and supernatants (extracellular toxin content) were transferred to LC vials and kept at −80 °C until UPLC-MS/MS analysis. Sample pellets were extracted with 1 mL of 80% methanol, vortexed (10s every 15 min, 4 times; DVX-2500 Multi-Tube Vortexer, VWR International, Pensylvania, USA) and centrifuged (12,470 × *g*, 10 min). Supernatant (intracellular toxin content) was transferred to LC vials and kept at −80 °C until UPLC-MS/MS analysis (Fig. S4).

#### 2.4.2 Extraction of toxins from aqueous samples

Water samples (100 mL) from sub-container 1 and 2 were combined and mixed. An aliquot of 30 mL (extracellular/dissolved toxin) was filtered through glass microfiber filters (55 mm; GF/C, GE Health Care, Buckinghamshire, UK) and kept at −80 °C until UPLC-MS/MS analysis (Fig. S4). Filters were transferred to 15 mL centrifuge tubes, extracted with 5 mL 80% methanol and vortexed (2500 rpm, 2 min; DVX-2500 Multi-Tube Vortexer). Extracts (intracellular/particulate associated toxin) were filtered (0.2 μm nylon syringe filters) and kept at −80 °C until UPLC-MS/MS analysis (Fig. S4).

#### 2.4.3 Extraction of free toxins from mussel tissue samples

Mussels (n = 10; 5 from each sub-container 1 and 2) were shucked and drained to separate the tissue and the extrapallial fluid. The extrapallial fluid was collected by opening the valve and inserting a needle fitted on a syringe between the mantle and shell. This fluid was centrifuged (12,470 × *g*, 10 min) and the supernatant was kept at −80 °C until UPLC-MS/MS analysis (Fig. S4). The drained tissue was blended into a smooth homogenous paste, by pulse blending four times for 15 s (5 s rest between pulses) (Waring Commercial, USA). Homogenate tissue (2.00 ± 0.05 g) was extracted with 8 mL of 80% methanol, vortexed (2300 rpm, 2 min; DVX-2500 Multi-Tube Vortexer) and centrifuged (2279 × *g*, 10 min). The supernatant was filtered and kept at −80 °C until UPLC-MS/MS analysis (Fig. S4).

#### 2.4.4 Extraction of toxins from faecal material

Collected faecal material (combined sample from sub-container 1 and sub-container 2) was centrifuged (2279 × *g*, 40 min; Sorvall, ST 40 R, ThermoScientific, USA) and the pellet was extracted with 5 mL 80% methanol, vortexed (2500 rpm, 2 min; DVX-2500 Multi-Tube Vortexer), centrifuged (12,470 × *g*, 20 min) and the supernatant was kept at −80 °C until UPLC-MS/MS analysis (Fig. S4).

### 2.5 Analysis by UPLC-MS/MS

MCs and NOD were analysed by UPLC-MS/MS (Waters, UK) as described previously (Turner et al., 2018). A summary of the operational parameters can be found in the Supplementary Material. Method validation parameters (specificity, linearity, limit of detection and quantification (LOD, LOQ), recovery, matrix effect, precision and ruggedness) were assessed to check that the analytical method was fit for purpose over an environmental relevant range of concentrations. The detailed validation study was reported in Turner et al. (2018).

### 2.6 Statistical analysis

Statistical analysis was made using GraphPad Prism version 8.2.1 (GraphPad Software, www.graphpad.com). Two-way ANOVA followed by Tukey’s multiple comparison test was used to determine potential presence of significant differences between concentrations at different time points. A statistically significant value of *p* = 0.05 was set.

## 3 Results and discussion

### 3.1 Quantification of cyanotoxins in feed stock culture

Both feed stock cultures of *M. aeruginosa* and *N. spumigena* used over 3 days of dosing were analysed by UPLC-MS/MS.

**Fig. 1:**
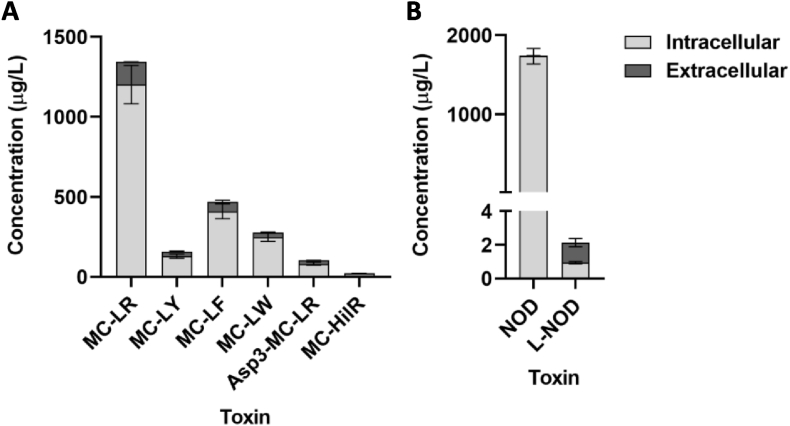
Concentration (μg/L) of intracellular and extracellular cyanotoxins produced by the cultures of *M. aeruginosa* (n = 3) (A) and *N. spumigena* (n = 3) (B) used for the exposure of *M. edulis*.

Six MC variant were present in the intracellular and extracellular phase of *M. aeruginosa* (1.1 × 10^8^ cells/L) (Fig. 1). The majority of MCs were contained within cells (90% of MC-LR and MC-LW, 88% of MC-LF and MC-HilR, 81% of MC-LY and 80% of [Asp3]-MC-LR/[Dha7]-MC-LR) whereas a small percentage was in solution. In decreasing order of abundance: MC-LR (1201.7 μg/L)>MC-LF (410.2 μg/L)>MC-LW (249.3 μg/L)>MC-LY (129.2 μg/L)>[Asp3]-MC-LR/[Dha7]-MC-LR (82.1 μg/L)>MC-HilR (20.4 μg/L).

NOD was present at 1734 μg/L (intracellular) and 5.9 μg/L (extracellular) and linear NOD (L-NOD) at 0.95 μg/L (intracellular) and 1.18 μg/L (extracellular) in the *N. spumigena* culture (1.2 × 10^8^ cells/L) (Fig. 1).

The production (%) of intracellular and extracellular cyanotoxins in *M. aeruginosa* and *N. spumigena* is in accordance with previous reports (Hameed et al., 2020; Jüttner and Lüthi, 2008).

### 3.2 Quantification of cyanotoxins in tank water

#### 3.2.1 Intracellular and extracellular toxins

**Fig. 2:**
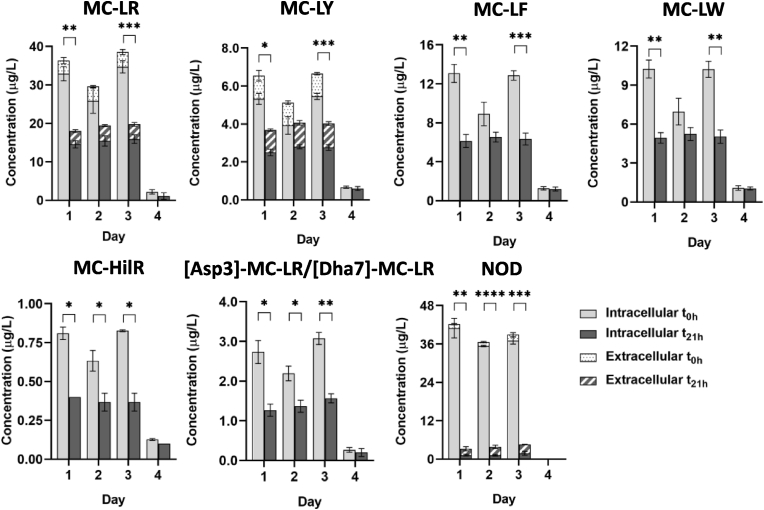
Concentration (μg/L) of intracellular and extracellular MCs (MC-LR, MC-LY, MC-LF, MC-LW, MC-HilR, [Asp3]-MC-LR/[Dha7]-MC-LR) and NOD detected in water tank at t_0h_ and t_21h_. Data is presented as mean and SD of n = 3. ∗*p* < 0.05, ∗∗*p* < 0.01, ∗∗∗*p* < 0.001, ∗∗∗∗*p* < 0.0001 t_0h_ vs t_21h_ based on two-way ANOVA followed by Tukey’s post-hoc test.

The clearance of cyanotoxins by *M. edulis* was monitored every 21 h during the 3 day exposure period (Fig. 2). No cyanotoxins were detected in the negative control containers.

Concentration levels and distribution of cyanotoxins detected in the exposure containers (t_0h_) were consistent with the cyanotoxin dose added (Table S1), except L-NOD which was <LOQ. On day 2 reported concentration levels of MCs after dosing (t_0h_) were lower than expected, potentially due to an extraction issue and more noticeable for the most hydrophobic MCs (MC-LY, MC-LF, MC-LW). Nevertheless, concentrations detected at t_21h_ were consistent across the 3 exposure days. Based on the added dose, 63–122% of cyanotoxins were detected in the water samples. Differences are attributed to the discrepancies on the extraction method used for the dosing stock solution and the water samples from the tank and the nature of the matrix (seawater). As shown by Lawton et al. (1994) and Coyle and Lawton (1996) a compromise has to be made to simultaneously extract a large number of toxins with different polarity. Statistically significant differences were observed between the total concentration (intracellular and extracellular) at t_0h_ and t_21h_ of MC-LR, MC-LY, MC-LF and MC-LY on day 1 and day 3 and of MC-HilR, [Asp3]-MC-LR/[Dha7]-MC-LR and NOD on day 1, 2 and 3 (Fig. 2).

Distribution showed that the majority of cyanotoxins were present intracellularly (Fig. 2). In addition, extracellular MC-LR (10.6%), MC-LY (20.0%) and NOD (3.61%) were present but at concentrations considerably lower than intracellular toxin content as expected based on toxin levels in “feed stock cultures”, which is representative of healthy cyanobacteria cultures. Extracellular concentrations of MC-LR, MC-LY and NOD at t_21h_ remained constant or slightly increased over the exposure period but changes were not statistically significant, except for MC-LR from day 1 to day 2 (*p* < 0.05) (Fig. S5).

No cessation of feeding was observed over the 3 exposure days indicating that *M. edulis* was not physiologically affected by the ingestion of these toxic cyanobacteria cultures.

Although feeding dose exposure lasted for 3 days, small quantities of intracellular MCs were detected on day 4 (Fig. 2), with concentrations similar after dosing (t_0h_) and after 21 h. Due to space limitations mussels were placed into their tanks between the daily water exchange and the following feeding dose (≈3 h). Consequently, it could be due to the release of cyanobacteria attached to the mussel shells or to the plastic container into the water. However, no clear justification was reached about the presence of traces of MCs on day 4.

#### 3.2.2 Clearance by *M. edulis*

Cyanobacterial clearance by *M. edulis* was evaluated by comparing the toxin content in water at t_0h_ and t_21h_ (Fig. S6). Most of the toxin content removed from the water by *M. edulis* accounted for cell-bound toxins (Fig. 2). No differences on the extracellular toxin content of MC-LR, MC-LY and NOD were observed between t_0h_ and t_21h_. The largest clearance was observed for NOD (mean removal 96.2%) whereas approximately half of the concentration of MCs remained in water after 21 h exposure over the 3 day exposure period (Fig. 2 and S6).

The higher removal of NOD by *M. edulis* could indicate the preference, the availability or the ease of filtering the filamentous *N. spumigena* than the unicellular *M. aeruginosa*.

In nature, cyanobacteria blooms occur mainly as colonies or filaments (Mur et al., 1999). However, lab studies on colonial cyanobacteria such as *Microcystis* show single cells instead, due to the absence of mucilage matrix, that could potentially hinder the ingestion by bivalves (Reynolds et al., 1981). It was reported that zebra mussels in presence of toxic and non-toxic colony forming strains of *M. aeruginosa* and the filamentous species *P. agardhii,* cleared the toxic filamentous species at a higher rate than the others but no clear explanation was given except of maybe differences in morphology or a range of undetermined cellular compounds (Dionisio Pires et al., 2005). Moreover, a recent study has shown that an increase in salinity affects the buoyancy of *N. spumigena* and at 32 psu the *N. spumigena* filaments tend to sink (Carlsson and Rita, 2019). Settling blooms of *N. spumigena* would make them more available to filter-feeders and a potential pathway for the transfer of NOD in the food chain (Carlsson and Rita, 2019; Mazur-Marzec et al., 2007).

### 3.3 Quantification of cyanotoxins in *M. edulis* tissue

#### 3.3.1 Accumulation

**Fig. 3:**
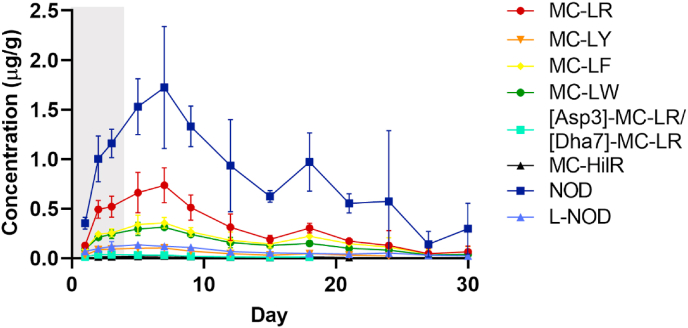
Temporal evolution of the concentration (μg/g) of cyanotoxins detected in *M. edulis* tissue over the 3 day exposure period (grey area) to a combined culture of *M. aeruginosa* and *N. spumigena* followed by a 27 day depuration period (white area). Data is presented as mean and SD of n = 3.

Toxins were accumulated in *M. edulis* flesh during the 3 days exposure period (Fig. 3). Results show that mussels accumulated a total of 0.77 μg/g of MCs and NOD (cyclic and linear) (except MC-HilR) within the first 24 h of exposure. Accumulation of toxins in the mussels increased by a factor of 2.82 on day 2 (2.18 μg/g) and of 3.15 on day 3 (2.44 μg/g) of exposure. The highest accumulation was observed for NOD>MC-LR>MC-LF≈MC-LW>L-NOD>MC-LY>[Asp3]-MC-LR/[Dha7]-MC-LR>MC-HilR and this trend was maintained over the following days. The toxin profile in mussel tissue was different to that observed in the combined culture of *M. aeruginosa* and *N. spumigena* (feed stock culture) (Fig. S7). Mussel tissue contained more NOD, L-NOD, MC-LY and MC-HilR and less MC-LR, MC-LF, MC-LW and [Asp3]-MC-LR/[Dha7]-MC-LR in comparison to the ratios in the feed stock culture. It was noted that in addition to NOD, the linearized form (L-NOD) was present in mussel flesh. L-NOD represented just ≈0.05% of the *N, spumigena* dose added to the tanks (Fig. S7). Bacterial degradation via a pathway that involves three hydrolysis enzymes (MlrA, MlrB and MlrC) and one oligopeptide transporter-like protein (MlrD) leads to the linearization of the cyclic MCs and NOD (Bourne et al., 2001; Feng et al., 2016; Massey and Yang, 2020). Consequently, this lead to a significant reduction in toxicity and facilitates the transport of the linearized peptides across the bacterial cell wall (Bourne et al., 2001). Thus, the presence of L-NOD at higher ratios in mussel tissue and extrapallial fluid (section 3.5) (Fig. S7) could indicate formation due to metabolism to reduce the toxicity and facilitate the transport across tissues. More research is needed to further understand the mechanisms involved in mussels.

The rapid accumulation observed in this study is consistent with previous studies even, when laboratory conditions were not comparable, (Kankaanpää et al., 2007; Svensen et al., 2005; Vasconcelos, 1995). Kankaanpää et al. (2007) which observed an accumulation of NOD if 0.70 μg/g dw in the digestive gland and of 0.10 μg/g dw in the remaining soft tissue of *M. edulis* within 24 h of being exposed to seawater containing *N. spumigena* (70–110 μg/L of NOD). Maximum concentrations of NOD up to 80.4, 1.90 and 250 μg/g dw accumulated in the body, gills and digestive gland of *M. edulis*, respectively, after 48 h exposure to *N. spumigena* (16 μg/L of NOD) were reported (Svensen et al., 2005). In Miller et al. (2010) 0.99 μg/g ww of MC-LR was accumulated in the gastrointestinal tissues in 24 h and after 21 d 97% was eliminated from the mussel (exposure was 10,600 μg/L of MC-LR in *Microcystis*).

During the depuration period, accumulation of toxins continued for the next 4 days reaching a maximum concentration on day 7 of 3.40 μg/g (sum of toxins) (Fig. 3). Over the following days accumulation of toxins in the mussels decreased until the total sum was 0.49 μg/g after 26 days of depuration. A slight increase of total concentration of toxins was observed after 15 days of depuration (1.76 μg/g). Demethylated MCs variants ([Asp3]-MC-LR/[Dha7]-MC-LR) were <LOD after 18 days of depuration, MC-HilR after 21 days and MC-LY after 24 days. The remaining toxins were partially eliminated as they were still found after 27 days spent in clean seawater.

The increase in detectable cyanotoxins in mussel tissue even after the removal of toxic cell source was previously reported. Andres et al. (2019) observed accumulation of paralytic shellfish toxins during the depuration period of *Perna viridis* which was exposed to cultures of *Alexandrium minutum* and suggested dead mussels or depuration products from other mussels as the source of toxins in that period. During our experiment, no mussel mortalities were found, thus, the accumulation observed on the 2nd, 4th and 15th day of depuration could be due to the toxins released by other mussels in the closed-system.

Other authors proposed the increase in detectable MCs and NOD concentrations during the depuration period to the release of bound-proteins (Amorim and Vasconcelos, 1999; Soares et al., 2004; Vasconcelos, 1995). In addition, differences in depuration of free and covalently bound MCs have been reported (Pham et al., 2017; Williams et al., 1997). Within 24 h free MC-LR eq was reduced by around 50% in clams (*Corbicula leana*) which had been exposed to *M. aeruginosa*. After seven days depuration MC concentration was <LOD. In contrast, covalently bound MC-LR eq remained constant for the first five days of depuration and were still detected on day ten of the depuration period (Pham et al., 2015).

Mussels sampled from the negative control tank showed no trace of toxins throughout the experiment.

### 3.4 Quantification of cyanotoxins in faecal material

Not all cyanobacteria present in the ecosystem are ingested, digested and ejected as faeces by filter-feeding organisms. Unwanted cyanobacteria may also be rejected as pseudo-faeces by embedding them in string of mucus for their disposal (Juhel et al., 2006). Selective grazing by filter-feeding organisms are not well known but the removal by faeces or pseudofaeces may act as a defence mechanism that would attenuate or reduce the hazards posed by these cyanobacteria.

No indication of pseudofaeces production was observed when *M. edulis* was exposed to a mixed cyanobacteria culture of *M. aeruginosa* (3.9 × 10^6^ cells/L) and *N. spumigena* (3.1 × 10^6^ cells/L).

**Fig. 4:**
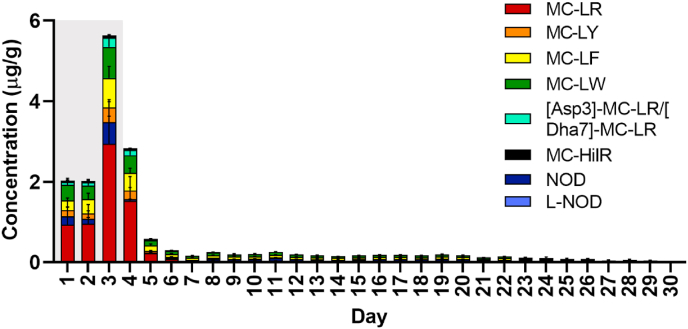
Temporal evolution of the concentration (μg/g) of cyanotoxins detected in faecal material of *M. edulis* produced over the 3 day exposure period (grey area) to a combined culture of *M. aeruginosa* and *N. spumigena* followed by a 27 day depuration period (white area). Data is presented as mean and SD of n = 3.

The excreted products during the exposure period contained MCs and NOD from day 1 (except L-NOD) (Fig. 4). Summed toxin concentrations were similar on day 1 and 2 but showed a steep increased from 2.01 μg/g on day 2–5.64 μg/g on day 3. The dominant cyanotoxins in the faecal material during the exposure period were MC-LR (≈49%), MC-LW (≈17%) and MC-LF (≈14%) (Fig. S7). The sum of remaining toxin concentrations represented <20% of the total content. The presence of toxins in the faeces implied that *M. edulis* actually ingested and digested the cyanobacteria during the exposure period. No cyanotoxins were detected in the negative control samples.

Following the end of the exposure period, the total concentration of cyanotoxins decreased from 2.83 μg/g on day 4 (1st depuration day) to 0.29 μg/g on day 6 (3rd day of depuration) (Fig. 4). Concentration levels then continued to decrease slowly to 0.02 μg/g over the next 24 days. During the first 3 days of depuration the dominant cyanotoxins were the same as in the exposure period. However, as depuration continued, differences in depuration rates between toxins were observed (Fig. S8). Overall, after 3 days of depuration most of the toxins were almost completely removed (100% [Asp3]-MC-LR/[Dha7]-MC-LR and MC-HilR > 99.0% MC-LR > 97.2% MC-LY > 95.9% NOD > 94.4% MC-LW > 93.1% MC-LF). At the end of the depuration period traces of MC-LR, MC-LW, MC-LY and NOD still remained in the faecal material.

In contrast to the present study, Amorim and Vasconcelos (1999) observed a steady increase in MC concentration (up to 150 μg/g dw) in faecal material during the first 3 days of depuration of *M. galloprovincialis* previously fed with *M. aeruginosa* (10^5^ cells/mL) for four days. This was followed by a steep decline of toxin levels until the end of the depuration period.

### 3.5 Quantification of cyanotoxins in extrapallial fluid of *M. edulis*

The extrapallial fluid could reach around 700–800 μL within an adult *M. edulis*. This aqueous microenvironment acts as bridge between the inner shell and the mantle epithelium (Zuykov et al., 2011).

**Fig. 5:**
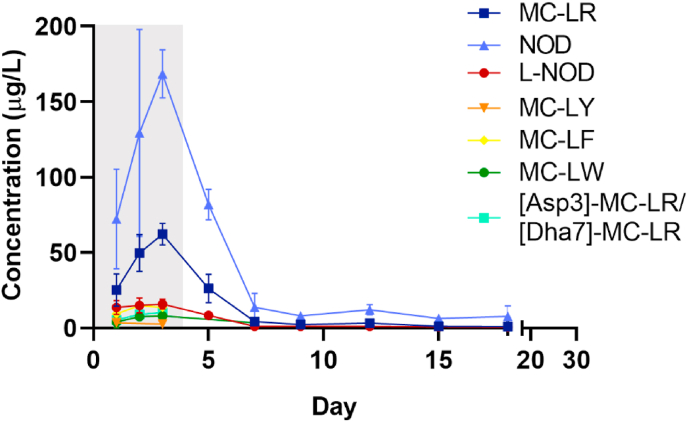
Temporal evolution of the concentration (μg/L) of cyanotoxins detected in extrapallial fluid of *M. edulis* produced over the 3 day exposure period (grey area) to a combined culture of *M. aeruginosa* and *N. spumigena* followed by a 27 day depuration period (white area). Data is presented as mean and SD of n = 3.

NOD and MC-LR were present at the highest concentration levels in the extrapallial fluid (Fig. 5). The distribution of cyanotoxins in extrapallial fluid was as follow: NOD>MC-LR>L-NOD>MC-LF>[Asp3]-MC-LR/[Dha7]-MC-LR>MC-LW>MC-LY, MC-HilR was <LOD (Fig. S7). Similar distribution was observed in the tank water for the most abundant toxins (Fig. S7). In contrast, L-NOD was detected at high concentration levels in the extrapallial fluid whereas it was <LOQ in the tank water. Concentration of MC-LR and NOD increased over the exposure time (3 days), reaching maximum concentrations of up to 168.5 μg/L of NOD and 62.3 μg/L of MC-LR on day 3, followed by a rapid decrease in concentration during the depuration period (Fig. 5). From day 9 (6th depuration day), concentrations of MC-LR and NOD were stable over time until day 18 (15th depuration day) when they reached the lowest concentrations detected and after that they remained <LOD. The minor toxins were detected only during the exposure time (day 1–3) except for day 7 (4th depuration day) on which MC-LW contributed to the overall toxin load.

Although the extrapallial fluid is an important bridge that participates in the detoxifying mechanism and exchange of metals between soft tissues and the inner shell of the mussel (Yen Le et al., 2020; Zuykov et al., 2011), no studies on the distribution of pollutants have been reported in the literature.

### 3.6 Determination of the cyanotoxin distribution within the experimental system

**Fig. 6:**
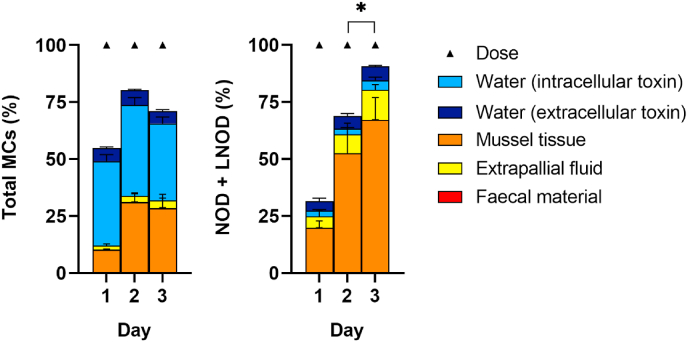
Recovery (%) of total MCs and NODs in different compartments (extracellular/dissolved and intracellular/particulate associated toxin in water, mussel tissue, extrapallial fluid and faecal material) based on the total amount of cyanotoxins (dose) added to the system during the 3 day exposure period to a combined culture of *M. aeruginosa* and *N. spumigena*. Data is presented as mean and SD of n = 3. ∗*p* < 0.05, day 1 vs day 2 vs day3 based on two-way ANOVA followed by Tukey’s post-hoc test.

When comparing the recoveries (%) of MCs and NOD in different compartments (extracellular/dissolved and intracellular/particulate associated toxin in water, mussel tissue, extrapallial fluid and faecal material) based on the total amount of cyanotoxins added to the system over the first 3 days, significant differences were observed between toxin analogues (Fig. 6). Distribution of MCs in the system was as follows: 39.3–46.4% in the water (33.9–39.8% intracellular toxin and 5.4–6.6% extracellular toxin), 10.2–28.3% in mussel tissue, 1.6–3.3% in extrapallial fluid and 0.1–0.3% in faecal material. An increase was observed from day 1 to day 2 and then remained stable or slightly lower in day 3. Between 19.7 and 45.1% of the total dose of MCs added was not recovered in the compartments analysed. In contrast to MCs, NOD showed a higher accumulation over 3 days (day 2–3 *p* < 0.05). NOD accumulated mainly in mussel tissue 19.8–67.1%, followed by extrapallial fluid 4.9–13.1%, water 6.7–10.9% (2.5–4.1% intracellular toxin and 4.1–6.3% extracellular toxin) and faecal material 0.01–0.06%. On day 1, 68.6% of the added dose was not located, which decreased to 9.3% on day 3 (Fig. 6).

**Fig. 7:**
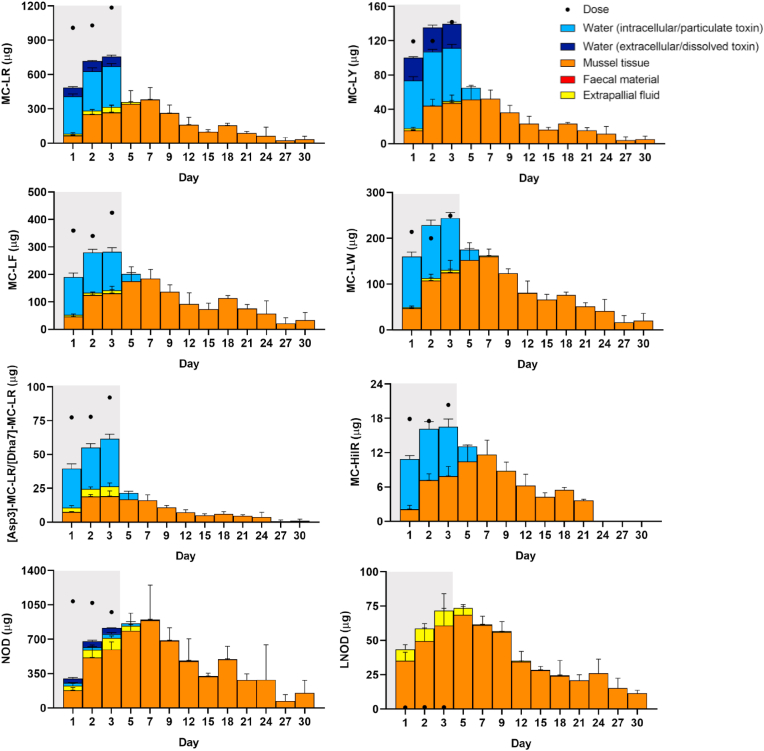
Amount (μg) of MCs (MC-LR, MC-LY, MC-LF, MC-LW, [Asp3]-MC-LR/[Dha7]-MC-LR and MC-HilR) and NODs (NOD and LNOD) detected in different compartments (extracellular/dissolved and intracellular/particulate associated toxin in water, mussel tissue, extrapallial fluid and faecal material) based on the total amount of cyanotoxins (dose) added to the system the 3 day exposure period (grey area) to a combined culture of *M. aeruginosa* and *N. spumigena* followed by a 27 day depuration period (white area). Data is presented as mean and SD of n = 3.

Among the cyanotoxins studied, a significant amount of MC-LR (30.3–51.8%), MC-LF (17.7–47.2%), [Asp3]-MC-LR/[Dha7]-MC-LR (29.3–49.0%), MC-HilR (7.99–39.3%) and NOD (16.5–72.6%) offered to the mussels was unaccounted for, especially on the first exposure day (Fig. 7).

Similar studies also reported gaps in the total burden of cyanotoxins in the experimental system (Dionisio Pires et al., 2005; Tencalla and Dietrich, 1997). Losses in the total cyanotoxin balance have been attributed to detoxification/biotransformation pathways after conjugation with amino acids and peptides, covalent binding to proteins, sample extraction/analysis protocols and biotic/abiotic degradation of toxins in the system.

Despite microbial or abiotic degradation, metabolic processes within *M. edilus* are more likely to have contributed to the unaccounted fraction of MCs and NOD. Conjugation with glutathione S-transferase, glutathione and cysteine are the most described MCs/NOD detoxification pathway in marine invertebrates (Gonçalves-Soares et al., 2012; Kondo et al., 1992; Lance et al., 2010). This process of detoxification increases the polarity of the conjugated product and enhances its excretion by the organism (Beattie et al., 2003; Kondo et al., 1992; Pflugmacher et al., 1998). Due to the lack of available analytical standards it was not possible to investigate the presence of potential MCs and NODs conjugates in the present study. However, in published studies MCs and NOD conjugates were detected and they represented an important fraction of the total cyanotoxin burden (Beattie et al., 2003; Galanti et al., 2013; Meissner et al., 2013; Metcalf et al., 2000; Pflugmacher et al., 1998; Zhang et al., 2009). Miles et al. (2016) showed that thiol-MCs conjugates are expected to be readily formed in vivo and to be potentially bioavailable both from free and conjugated forms.

Covalently-bound hepatotoxins might also represent a high fraction (Lepoutre et al., 2020; Williams et al., 1997) or a small fraction (Dionisio Pires et al., 2005; Pham et al., 2017) of the total cyanotoxin content in mussels. Hepatotoxins specifically target protein phosphatases involving a rapid and reversible binding followed by a covalent binding after several hours (Campos and Vasconcelos, 2010; Hastie et al., 2005; Maynes et al., 2006; Pereira et al., 2013). However, covalently-bound MCs are not considered toxic and doubts exist as to whether they would be biologically available to consumers (Ibelings and Chorus, 2007; Vasconcelos et al., 2001), unless they are released back as free toxins (Miles et al., 2016). So far, only the study of Smith et al. (2010) suggests that bound toxins are toxic and showed that proteolytic enzymes could free covalently-bound toxins.

To date, the accurate detection of covalently bound hepatotoxins remains a challenge. The main technique involves the Lemieux oxidation of the Adda moiety to form 2-methyl-3-methox-4-phenylbutiric acid (MMPB) which can be monitored by MS, UV, fluorescence or flame ionisation detection (Brown et al., 2018; Foss and Aubel, 2015; Neffling et al., 2010; Sano et al., 1992; Williams et al., 1997). The MMPB technique (Foss and Aubel, 2015) detects MCs/NODs indiscriminately so individual MC congeners or NOD cannot be identified. In addition, discrepancies have been reported due to low recoveries during the oxidation procedure, additional concentration/clean-up steps especially when complex matrices are encountered, detection of potential metabolites and microbial degradation products being detected and/or signal suppression (Brown et al., 2018; Cadel-Six et al., 2014; Neffling et al., 2010).

Even if covalently-bound hepatotoxins remained undetected by the applied protocols and actual concentrations of MCs and NODs were higher than the ones reported in this study, free toxins may be a better reflection of levels and dynamics of intracellular toxins in water. Lepoutre et al. (2020) compared the free and protein-bound MCs accumulation by two bivalve species (*Anodonta anatina* and *Dreissena polymorpha*) to evaluate which one best reflects levels and dynamics of MC-producing cyanobacteria in water. They showed that free MC in bivalve tissues highlight better the dynamics of intracellular MC in water. As bound MC remain longer than free MC in tissues after the exposure time (Lance et al., 2010, 2016; Lepoutre et al., 2020) total MC would not represent short-term environmental MC variations.

Conversely, some studies still reported a large gap in the cyanotoxin balance sheet for mussel experiments even when detoxification and covalently bound MC were taken into account (Dionisio Pires et al., 2005; Tencalla and Dietrich, 1997). As far as we know, Williams et al. (1997) which reported covalently bound MC, this is the only study that was able to trace all of the MC added to the system.

We could only trace part of the amount of MCs and NODs administered in the experiment (Fig. 7). Data revealed that L-NOD was present at high amounts during the exposure (34.9–60.8 μg in mussel and 8.46–10.9 μg in extrapallial fluid) and depuration (11.5–68.5 μg in mussel and 0.36–5.07 μg in extrapallial fluid) period even when the dose of L-NOD added was significantly lower (1.16–1.41 μg) (Fig. 7). L-NOD represented 5–35% of that of NOD in mussel tissue and from 11 to 30% in extrapallial fluid. The presence of L-NOD only in mussel and extrapallial fluid could indicate that it was produced due to metabolism within the mussels to reduce the toxicity and facilitate the transport across tissues.

Demethylated MCs were the only toxins that did not accumulated in mussel tissue during the depuration period.

### 3.7 Health risk

With increased awareness of multiple sources of human exposure to cyanotoxins and the public health risks associated with them, the WHO has released guidelines for cyanotoxin exposure (WHO, 2017). The WHO set a provisional tolerable daily intake (TDI) of 0.04 μg/kg body weight (bw) for MC-LR. The provisional TDI was calculated for lifetime exposure based on a 13-week study with mice to which the MC-LR dose was administered orally by gavage and applying an uncertainty factor of 1000 to account for intra-species and inter-species variability and in particular due to lack of data on chronic toxicity and carcinogenicity. Despite its conservative approach, it is limited to MC-LR and does not consider differences in toxicities of other MCs or likely scenarios as single dose or exposure for several weeks during the cyanobacterial bloom. To address differences in toxicity among MCs analogues some studies suggest to use the toxicity equivalency factor (TEF) approach which estimates the toxicity/potency of a MC analogue relative to the toxicity/potency of MC-LR (Altaner et al., 2020; Garibo et al., 2014; Ikehara et al., 2009). However, the relative toxicities determined by *in vitro* (Fischer et al., 2010; Garibo et al., 2014; Ikehara et al., 2009; Niedermeyer et al., 2014), *in vivo* (Chen et al., 2006; Chernoff et al., 2020) or *in silico* predictions studies (Altaner et al., 2020) are not consistent. Given the poor knowledge of mechanism of action and non-standardised toxicity studies based on single doses, intraperitoneal injections instead of oral administration and single toxins instead of multiple toxins, it is therefore difficult to define a TEF for the different MC variants. To consider differences in exposure times, Ibelings and Chorus (2007) derived an additional acute tolerable intake (TI) of 2.5 μg/kg bw and seasonal TDI of 0.4 μg/kg bw.

**Fig. 8:**
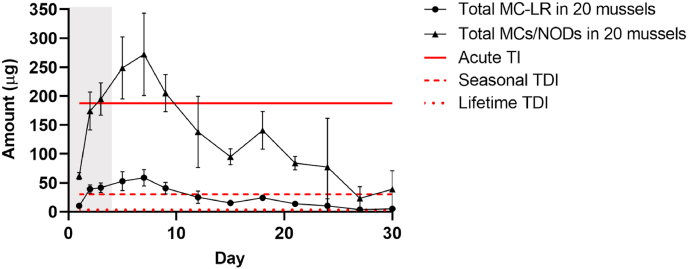
Amount (μg) of MC-LR and total MCs/NODs accumulated in *M. edulis* tissue over the 3 day exposure period (grey area) to a combined culture of *M. aeruginosa* and *N. spumigena* followed by a 27 day depuration period (white area). Data is presented as mean and SD of n = 3. Estimated tolerable limits (μg) (acute tolerable intake (TI), seasonal tolerable daily intake (TDI) and lifetime TDI) of MC-LR based on a body weight of 75 kg and WHO (2017) and Ibelings and Chorus (2007).

Fig. 8 shows the comparison of the amount of free toxin the average adult (default body weight 75 kg) would be exposed to, based on a typical portion size (≈80 g) of mussels (The European Food Safety Authority estimated 400 g of shellfish meat as a large portion size for risk assessment purposes, EFSA (2010)) and the concentrations of toxins reported in this study to determine if human consumers would exceed the acute, seasonal and lifetime TDI. Based on the free MC-LR content in *M. edulis* tissue risks would be associated to lifetime TDI during the exposure and the depuration period and to the seasonal TDI during the exposure period (day 2 and 3) and during the following 9 days of depuration (Fig. 8). Although the WHO set up the TDI limit only for MC-LR, this study showed that *M. edulis* accumulated considerable amounts of other MCs variants and NOD after exposure of *M. aeruginosa* and *N. spumigena* which should be taken into account (Fig. 3). Assuming that all MCs/NODs have equivalent toxicity to MC-LR, based on the total free MCs/NODs accumulated in *M. edulis*, the estimated acute TI limit was exceeded by the end of the exposure period (day 3) and at the beginning of the depuration period (day 5–9). Whereas the estimated seasonal and lifetime TDI limits were far exceeded on day 7 (maximum amount of MCs/NODs accumulated in *M. edulis*) by a factor of 9 and 90, respectively. In addition, eating a portion of 20 mussels even after 27 days of depuration would still result in the seasonal and lifetime TDI being exceeded by a factor of 1.3 and 13, respectively. Therefore, assuming that all MCs/NODs have equivalent toxicity, consuming mussels harvested during or even shortly after HABs, in close proximity to shellfish beds, carries a high health risk.

These results, however, should be interpreted with caution. Currently, there is no regulatory monitoring of freshwater toxins in marine shellfish and many factors should be addressed when considering regulatory limits on shellfish, including different toxicity of MCs analogues, multiple/simultaneous toxin exposure, effects on non-target organisms, mechanism of action of each toxin and multiple exposure scenarios, among others.

## 4 Conclusions

Resembling a natural freshwater and brackish harmful cyanobacteria bloom inputting into the coastal environment, showed that MCs/NODs rapidly accumulated in the common blue mussel *M. edulis.* Maximum concentrations (3.40 μg/g) were reported in mussels 4 days after the cessation of the toxic cell source indicating the potential release of bound and/or conjugated MCs/NODs. Depuration was slow and incomplete with total cyanotoxin concentration remaining in mussel flesh up to 0.49 μg/g even after 27 days. Distribution profiles of MCs/NODs within different parts of the experimental system (feed stock culture, water, mussel tissue, faecal material and extrapallial fluid) varied. In terms of toxin burden, MC-LR and NOD contributed similarly to the total content in the feed stock culture, whereas in mussel tissue and extrapallial fluid NOD (46% and 57%, respectively) was the most abundant and in faecal material it was MC-LR (49%) and the other MCs variants. Interestingly, the presence of L-NOD only in mussel and extrapallial fluid would indicate that it was a product of metabolic processes. Assessing the toxin budget within the experimental system has proven to be a highly complex process and a fraction of toxins was unaccounted for.

The rapid accumulation and slow depuration of MCs/NODs in *M. edulis* might represent a high risk to wildlife and the general public and highlights the need for monitoring of multiple co-occurrence of marine and freshwater cyanotoxins in bivalves obtained from areas potentially exposed to cyanobacteria. In addition, current regulatory guidelines assume all MC variants are equally toxic, do not consider the co-occurrence of multiple toxins and they were based on sub-chronic exposure data on mice. Consequently, they may overestimate/underestimate the risk of hepatotoxins to human health. Many exposure patterns should also be taken into account to estimate risk as likely scenarios could involve acute exposure due to consumption of highly contaminated shellfish in a single meal or chronic exposure during a several-week cyanobacterial bloom.

## Author contributions

Dolores Camacho-Muñoz: Writing – original draft, Writing – review & editing. Julia Waack: Investigation, Validation, Writing – original draft, Writing – review & editing. Andrew D. Turner: Conceptualization, Funding acquisition, Methodology, Project administration, Resources, Supervision, Validation, Writing – review & editing. Adam M. Lewis: Conceptualization, Funding acquisition, Methodology, Project administration, Resources, Supervision, Writing – review & editing. Linda A. Lawton: Conceptualization, Funding acquisition, Methodology, Project administration, Resources, Supervision, Writing – review & editing. Christine Edwards: Conceptualization, Funding acquisition, Methodology, Project administration, Resources, Supervision, Writing – review & editing.

## Declaration of competing interest

The authors declare that they have no known competing financial interests or personal relationships that could have appeared to influence the work reported in this paper.
