# Supplementary material for: Rapid uptake and slow depuration: Health risks following cyanotoxin accumulation in mussels?
Source: Environ Pollut. 2021 Feb 15;271:116400. doi: 10.1016/j.envpol.2020.116400 (PMC7859834; doi:10.1016/j.envpol.2020.116400)
Supplement: Multimedia component 1 [file mmc1.docx]

**SUPPLEMENTARY MATERIAL**

**Rapid uptake and slow depuration: marine pollution with freshwater cyanotoxins?**

**Dolores Camacho-Muñoz^a*^, Julia Waack^a,b^, Andrew D. Turner^b^, Adam Lewis^b^, Linda A. Lawton^a^, Christine Edwards^a^**

^a^School of Pharmacy and Life Sciences, Robert Gordon University, Aberdeen, AB10 7GJ, UK

^b^Centre for Environment, Fisheries and Aquaculture Science, Barrack Road, The Nothe, Weymouth, Dorset, DT4 8UB, UK

**UPLC-MS/MS**

UPLC conditions

MCs and NOD were quantified by UPLC-MS/MS (Waters, UK). A Waters Acquity UPLC system coupled to a Waters Xevo TQ tandem quadrupole mass spectrometer was used (Manchester, UK). Separation was carried out on a Waters Acquity UPLC BEH C18 column (50 x 2.1 mm; 1.7 µm particle size) in conjunction with a Waters VanGuard BEH C18 guard cartridge (5 x 2.1 mm; 1.7 µm particle size) at 60°C. Mobile phase was acetonitrile with 0.025% FA (B) and water with 0.025% FA (A) at a flow rate of 0.6 mL/min. Gradient elution was as follows: 2% B initial conditions rising to 25% B at 0.50 min, holding until 1.5 min, rising to 40% B at 3.0 min, increasing further to 50% B at 4 min, a quick rise to 95% B at 4.1 min, holding until 4.5 min, dropping back to 2% B at 5 min and holding until 5.5 min. Samples were kept at 10°C. Injection volume was 5 µL. Each instrumental sequence started with a series of injections of blanks, followed by toxin calibration standards and by and extract of RM-BGA as a matrix-based retention time marker. At the end of each instrumental sequence column was flushed with a mixture of water/acetonitrile at 60°C followed by a second flush at 30°C.

MS/MS conditions

The Xevo TQ (Waters, UK) was operated in positive ESI mode. Tune parameters were as follows: 1.0kV capillary voltage, 150°C source temperature, 600°C desolvation temperature, 600 L/h desolvation gas flow, 0.15 mL/min collision gas flow. Selected reaction monitoring (SRM) transitions and cone and collision voltages were optimized by infusion of pure standards prepared in mobile phase starting conditions. Good separation was obtained for all toxins except for [Asp3]-MC-LR and [Dha7]-MC-LR, which shared the same SRM transitions and therefore they were reported together. Acquisition and processing of MS data was done using MassLynx v 4.1 software (Waters). Quantification was carried out by external calibration using certified reference standards over the range 0.6-170 ng/mL.

| **Toxin** | **SRM transitions** | **Cone (V)** | **Collision energy (eV)** |
| --- | --- | --- | --- |
| MC-RR | 519.9>134.9; 126.9 | 30 | 35; 50 |
| LNod | 692.0>135.0; 107.0 | 55 | 54;58 |
| Nod | 825.5>135.1; 103.1 | 55 | 60; 100 |
| MC-LA | 910.1>135.1; 106.9 | 35 | 70; 80 |
| [Dha7]-MC-LR | 981.5>135.0; 106.8 | 75 | 75; 80 |
| [Asp3]-MC-LR | 981.5>134.9; 106.9 | 75 | 70; 80 |
| MC-LF | 986.5>213.0; 135.0 | 35 | 60; 65 |
| MC-LR | 995.6>135.0; 127.0 | 60 | 70; 90 |
| MC-LY | 1002.5>135.0; 106.9 | 40 | 70; 90 |
| MC-HilR | 1009.7>134.9; 126.9 | 75 | 75; 90 |
| MC-LW | 1025.5>134.9; 126.8 | 35 | 65; 90 |
| MC-YR | 1045.6>135.0; 126.9 | 75 | 75; 90 |
| MC-HtyR | 1059.6>134.9; 106.9 | 75 | 70; 90 |
| MC-WR | 1068.6>134.9; 106.9 | 80 | 75; 100 |

**Table S1.** Recoveries of intracellular MCs and NODs (µg/L) in water samples detected immediately after feeding on day 1, 2 and 3 compared to values detected in feed stock cultures.

| **Cyanotoxins** | **Day** | **Intracellular**  **concentration**  **(µg L^-1^)** | **Feed stock intracellular concentration**  **(µg L^-1^)*** | **Recovery (%)** |
| --- | --- | --- | --- | --- |
| **NOD** | 1 | 41.0 | 48.4 | 85 |
|  | 2 | 35.4 | 47.5 | 75 |
|  | 3 | 37.1 | 43.5 | 85 |
| **MC-LR** | 1 | 32.9 | 40.0 | 82 |
|  | 2 | 25.9 | 40.9 | 63 |
|  | 3 | 34.7 | 47.8 | 73 |
| **Asp^3^-MC-LR/ [Dha^7^]-MC-LR** | 1 | 2.73 | 2.71 | 101 |
|  | 2 | 2.20 | 2.76 | 80 |
|  | 3 | 3.07 | 3.33 | 92 |
| **MC-HilR** | 1 | 0.81 | 0.69 | 117 |
|  | 2 | 0.64 | 0.68 | 94 |
|  | 3 | 0.82 | 0.81 | 101 |
| **MC-LY** | 1 | 5.33 | 4.37 | 122 |
|  | 2 | 3.92 | 4.31 | 91 |
|  | 3 | 5.46 | 5.16 | 106 |
| **MC-LF** | 1 | 13.1 | 13.9 | 94 |
|  | 2 | 8.91 | 13.5 | 66 |
|  | 3 | 12.9 | 16.6 | 77 |
| **MC-LW** | 1 | 10.2 | 8.56 | 119 |
|  | 2 | 6.96 | 8.12 | 86 |
|  | 3 | 10.2 | 10.0 | 102 |

***-** Based on intracellular cyanotoxins detected in feed stock cultures and their expected

theoretical concentration in tank water according to the dilution applied

**Fig. S1.** Chemical structure of microcystins variants with variable X and Y substituents denoting the specific analogue and nodularin (linear and non-linear) included in this study. Adda (3-amino-9-methoxy-10-phenyl-2,6,8-trymethyldeca-4,6-dienoic acid); isoGlu (isoGlutamic acid); Mdha (methyl-dehydro-alanine); isoAla (iaoAlanine); isoMe-Asp (isomethyl aspartic acid); Mdhb (methyl-dehydro-butyrine; Ala: alanine; Arg (arginine); Leu: leucine; Me: methyl; Phe: phenylalanine; Tyr: tyrosine; Trp: tryptophan;

**Fig. S2.** Schematic illustration of exposure and control tanks. Each tank was filled with 10 L of filtered seawater housing 70 mussels (*M. edulis*) supported on mesh baskets. Exposure tanks received a dose of 300 mL of *N. spumigena* (≈0.52 mg NODs) and 400 mL of *M. aeruginosa* (≈0.95 mg MCs) and were fed with shellfish diet for the first 3 days (accumulation period). Control tanks were fed with shellfish diet during the accumulation period. Both exposure and control tanks were exclusively fed with shellfish diet during the following 27 days (depuration period).

**Fig. S3.** Schematic illustration of the sampling procedure of exposure tanks. Subtanks 1 and 2 were treated identically in terms of mussel diet. Samples taken from subtanks 1 and 2 were combined and treated as one. UPLC-MS/MS: Ultrahigh performance liquid chromatography coupled to tandem mass spectrometry.

**Fig. S4.** Summary of sample extraction protocols used in this study. MeOH: methanol, UPLC-MS/MS: Ultrahigh performance liquid chromatography coupled to tandem mass spectrometry.

**Fig. S5.** Concentration (µg/L) of intracellular and extracellular MCs (MC-LR, MC-LY, MC-LF, MC-LW, MC-HilR, [Asp3]-MC-LR/[Dha7]-MC-LR) and NOD detected in water tanks at t_0h_ and t_21h_. Data is presented as mean and SD of n=3. **p*<0.05, *****p*<0.0001 day 1 vs day 2 vs day 3 based on two-way ANOVA followed by Tukey’s post-hoc test.


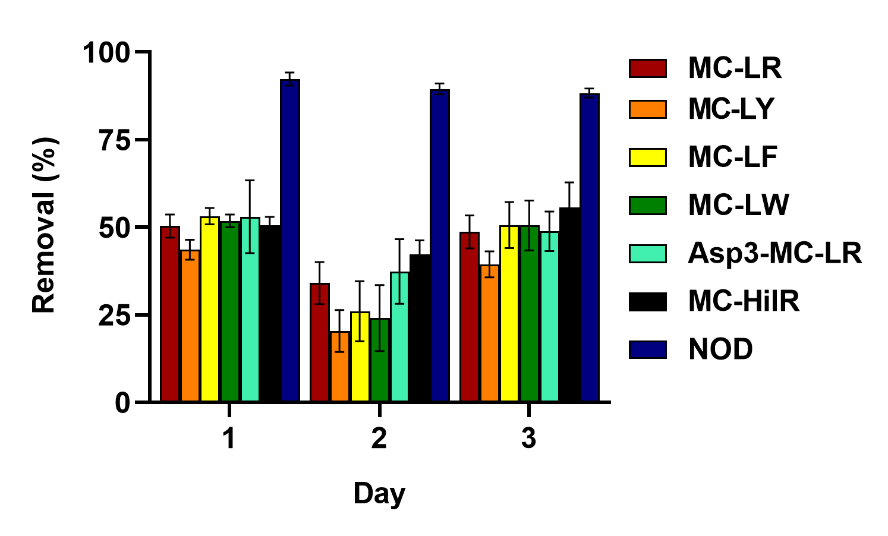


**Fig. S6.** Clearance (%) of cyanotoxins by *M. edulis* detected in tank water over 21h during the 3 day exposure period. Data is expressed as mean and SD of n=3.

**
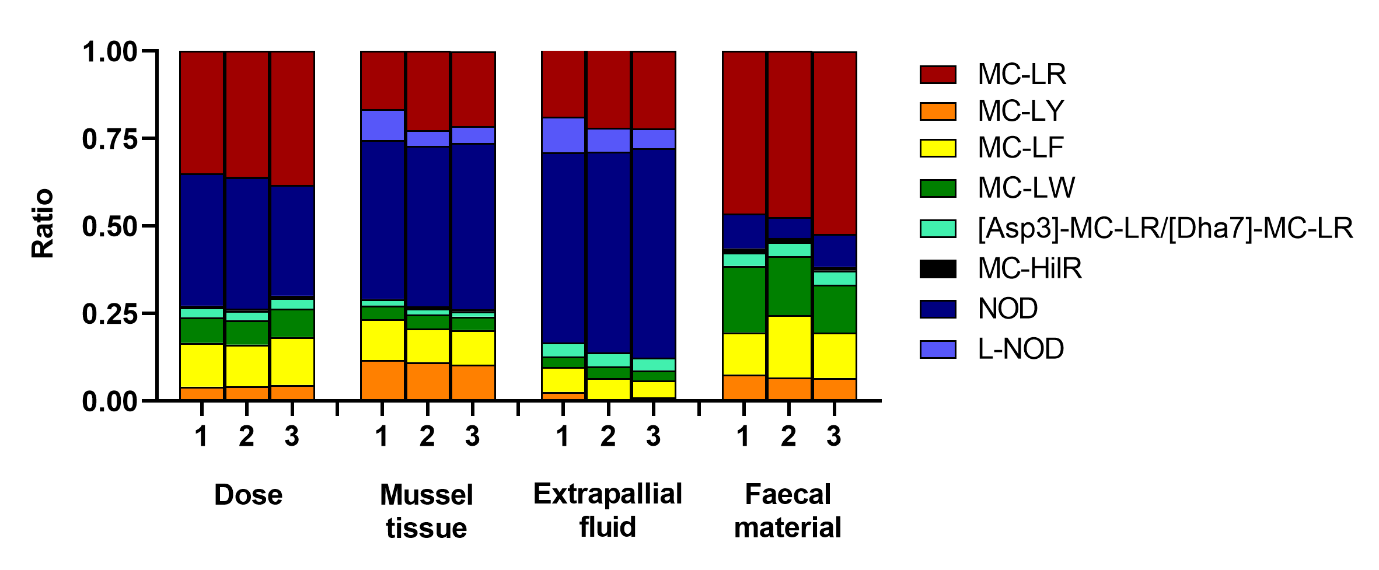
**

**Fig. S7.** Distribution profile of cyanotoxins in the feed stock dose, mussel tissue, extrapallial fluid and faecal material on the 3 days of exposure period.

**Fig. S8.** Depuration rate (%) of cyanotoxins over 27 days in the faecal material.
